# Repellent and Attractant Activities of Organic Compounds on Female and Male *Philonthus decorus* (Coleoptera, Staphylinidae)

**DOI:** 10.3390/biology13050294

**Published:** 2024-04-25

**Authors:** Liudmyla Faly, Viktor Brygadyrenko, Algimantas Paulauskas

**Affiliations:** 1 Research Institute of Natural and Technological Sciences, Department of Biology, Faculty of Natural Sciences, Vytautas Magnus University, 44248 Kaunas, Lithuania; 2 Department of Zoology and Ecology, Faculty of Biology and Ecology, Oles Honchar Dnipro National University, 49010 Dnipro, Ukraine; brigad@ua.fm; 3 Department of Parasitology, Veterinary and Sanitary Expertise, Faculty of Veterinary Medicine, Dnipro State Agrarian and Economic University, 49600 Dnipro, Ukraine

**Keywords:** insect repellents, insect attractants, organic compounds, fuel mixes, solvents, locomotor activity repellent activity, attractant activity of insects

## Abstract

**Simple Summary:**

The negative impact of numerous chemical compounds entering the environment from various anthropogenic sources is one of the reasons for the decline in populations and biodiversity of aboveground invertebrates. Chemical compounds can cause various effects (attracting or repelling) on insects. In laboratory conditions, the motor response of 1802 adult *Philonthus decorus* Gravenhorst beetles to 40 organic compounds and mixtures of compounds (acids, alcohols, ketones, phenols, aldehydes, aromatic carbohydrates solvents, and vehicle fuels) was revealed. It has been established that females of this rove species are generally less sensitive to odors. Organic acids in most cases were characterized by a repellent (oleic, acetic, oxalic, citric, formic acids) or neutral effect on beetles. The exception was some amino acids that have a moderate attractive effect. Alcohols most often exhibited the properties of moderate repellents or neutral substances for *Ph. decorus* imagoes. The exceptions were butyl alcohol (strong repellent of females), and methyl alcohol (moderate attractant of females). Aldehydes showed a moderate repellent effect on males and did not affect females. Aromatic hydrocarbons had a weak repellent effect on rove beetles. Organic solvents and fuels exerted no repellent or attractant effects on *Ph. decorus*. In most cases, they had a moderate repellent or neutral effect on these insects, with the exception of diesel fuel. This type of fuel attracted females. The majority (55%) of the chemical compounds and mixtures of compounds participating in the experiment had no attractant or repellent effect on the staphylinids.

**Abstract:**

The use of organic compounds in different spheres of human activity is accompanied by their influx to and accumulation in the environment. The negative impact of those compounds can be one of the reasons for a decline in populations and biodiversity of aboveground invertebrates. Chemical compounds can potentially cause a variety of effects (attractant or repellent) on insects, including species of the Staphylinidae family. In a laboratory experiment, we identified repellent and attractant influence of 40 organic compounds and mixtures of compounds (acids, alcohols, ketones, phenols, aldehydes, aromatic carbohydrates solvents, and vehicle fuels) on *Philonthus decorus* Gravenhorst, 1802. The ambulatory responses of the males and females to the same chemical compounds most often varied. A strong repellent activity against both sexes of *Ph. decorus* was caused by oleic acid, while hexane repelled the males. Acetic acid, 1-butanol, and ammonia solution were found to be strongly repellent against females. A moderate (average) repellent activity towards male *Ph. decorus* was displayed by organic solvents and fuels, some alcohols (isopropanol, isoamyl alcohol, methanol, ethanol), acids (acetic, formic acid), aromatic carbohydrates (toluene, xylene), and formaldehyde. Female *Ph. decorus* in general were less sensitive to the odors. The list of repellents with moderate activity against the females was much shorter: solvent 646, white spirit, toluene, isopropanol, isoamyl alcohol, citric and oxalic acids, and glycerol. Moderate attractant activity for *Ph. decorus* was exhibited by some amino acids, alcohols, and fuel mixes: glycine and L-cysteine (for the males), and phenylalanine, methanol, and diesel fuel (for the females). The rest of the 40 chemical compounds we studied caused no ambulatory responses in *Ph. decorus*. The difficulties we encountered in the interpretation of the results suggest a need for further experimental studies that would expand the knowledge of the chemoecology of insects.

## 1. Introduction

The human brain perceives and analyzes data from the environment using analyzing systems. The leading analyzers are visual, processing over 60% of the obtained data, and 35% are auditory. The percentages of the data processed by the analyzers of insects are different. Approximate characteristics were found at the level of hypotheses [1,2].

Receptors that are sensitive to certain irritants are responsible for the perception of chemical signals in the sensory system. The chemical signals are typically perceived by insects by the antennae (more rarely by the maxillary and labial palpi, the tarsi of the front legs, or other body parts), which bear the olfactory sensilla of various functional types. The olfactory reception in insects includes the detection of aromatic substances by the chemoreceptors, transmission of information, and its processing in the central nervous system. In response, there form locomotor commands that coordinate a number of behavioral reactions—feeding-related, sexual, and social [3,4]. Insects have a complex sophisticated system of chemoreception which, besides the perception of odor, is responsible for taste, and also chemical communication between individuals. It allows insects to find even very low concentrations of certain odors and recognize food objects, individuals of their species, including those of opposite sex, potential enemies, etc. [5,6]. The diversity of chemical compounds вeщecтв in the environment caused the emergence of a large spectrum of chemoreceptors of various specificity in insects. Those receptors are represented by several classes of proteins and are encoded by hundreds of genes. 

This communication takes place through semiochemicals, for example, pheromones: releasers; primers; sexual, aggregational (pheromones of unification), signaling, and trail pheromones; and others [7,8,9]. 

Other types of semiochemicals include allelopathic compounds, substances that have an effect on individuals of another species, such as the inhibition of the development of others, and allomones, substances that are beneficial for an organism that produces them by affecting other organisms. Allomones can potentially attract (flower odor, nectar, which attract insects) or repel (protective secretions of plants and animals). Attractants of insects have been studied in detail since their discovery in the mid-19th century. The first synthesized attractants emerged later, in the 1970s. Until now, those substances have been successfully used to attract beneficial species and combat pests. Repellents, on the other hand, fend off insects [10,11]. Those natural and synthetic compounds are used to divert pests of food storages, blood-sucking arthropods, including for the purpose of prophylaxis of infectious diseases. Their advantage over insecticides is that they act selectively and reduce harm to non-target species. The combined use of attractants and insecticides in glue traps allows the preservation of beneficial insects in agrocoenoses. 

The intensified use of organic compounds in various industrial spheres, e.g., agriculture and forestry, and in households ultimately leads to the accumulation of those compounds in the environment. Therefore, the effects they have on populations of natural fauna must be evaluated. Against the backdrop of intensive development of new technologies and urbanization, epigean insects are subject to dozens, and maybe hundreds of various chemical compounds: organic solvents, acids, food additives, and many others. The main sources of technogenic emission of organic compounds into the environment are power plants, chemical and food enterprises, and also vehicles [12,13]. For example, the chemical industry alone, which transforms natural raw material into a large amount of various chemical products, encompasses inorganic and organic chemistry, petrochemistry, agrochemistry, silicates, polymers, pharmaceutical chemistry, perfumery, and cosmetics. The influx of those compounds into the environment is associated with processes of burning and recycling of organic raw materials [14]. Organic compounds and their mixtures, used by people for various purposes, can potentially be attractive or repellent to insects. This may be one of the reasons why the populations and biodiverisity of many groups of beneficial and rare insects are declining. As of now, data about effects of organic compounds on arthropods are fragmented [15,16].

A group of invertebrates that is particularly hard to study is Staphylinidae, predatory insects that lead a secretive lifestyle. The best-researched genus of this family regarding behavioristic reactions to various compounds is *Aleochara*, species of which are used in biological pest management. For instance, larvae of those beetles parasitize puparia of Diptera and Tenthredinidae [17,18]. Identifying the effects of organic compounds with the potential to repel or attract common or rare staphylinids is possible only through experiments. Also, based on behavioral reactions of staphylinids, it is possible to identify compounds and mixtures that are dangerous to them. 

The genus *Philonthus* Stephens, 1829 (=*Spatulonthus* Tottenham, 1955) is cosmopolitan and diverse. The global fauna comprises over 1200 described species, about 100 of them in Europe. *Philonthus decorus* (Gravenhorst, 1802) is a European species, distributed from the British Isles and Northern Fennoscandia to the Pyrenees and Northern Balkans. In the territory of Lithuania, it is a common dweller of natural forest ecosystems which lives in forest litter and can occur in different types of decomposing substrates [19,20].

The objective of this study was to experimentally identify the repellent and attractant influence of some organic compounds and their mixtures for males and females of *Ph. decorus* in laboratory conditions. 

## 2. Materials and Methods

A laboratory culture of *Ph. decorus* was kept in transparent plastic containers (50 × 30 × 20 cm) at the temperature of 21–23 °C and the relative air humidity of 50–55%. The males and females were kept separately (50 individuals in each container). As a substrate we used moistened sand. The beetles were collected using Barber pitfall traps in forest ecosystems around the city of Kaunas (Lithuania). 

To experimentally study effects of various organic compounds on the locomotor activity of *Ph. decorus*, a wooden carcass with no bottom was constructed. The construction consisted of two long lateral planks (150 cm long and 10 cm height), connected with beams (10 cm long and 10 cm tall) on the ends. The area of the experimental ground was 150 × 10 cm. On the laboratory table, beneath the carcass, a strip of paper was placed with parallel lines after every 10 cm. Therefore, the migratory field for the beetles comprised 15 squares (10 × 10 cm), arranged in one line. Prior to each experiment, inside the wooden carcass we put thick transparent polyethylene. The changeable insert was fixed using duct tape on both sides. For each experiment, we used new polyethylene for removal of the influence of pheromone traces on the polyethylene on the results of the experiment. To limit the evaporation and preserve the concentrations of volatile organic compounds before each experiment, this construction was covered by two glass panels, closely connected in the center. Inside the carcass (the marginal left square sized 10 × 10 cm) we put a plastic lid with an attached cotton swab, moistened by one drop (0.05 mL) of tested compound or mixture of compounds. This dose was used because it is easy to measure and it is optimal regarding the volume of air in the experimental chamber (15 dm^3^). The glass panels were moved sideways from the center and 50 specimens of *Ph. decorus* of a certain sex were placed in the center of the 8th 10 × 10 section. Then, the glass panels were put together again and the videorecording of the experiment was started. The beetles could stay where they were, start running to the left (towards the odor) or to the right (away from the source of the odor). The video was recorded using a smartphone video camera, fixed at the height of 1 m above the experimental chamber. According to the movement direction of the staphylinids, we identified the reaction of the beetles to the tested compounds or their mixtures (repellent or attractive). We tested 40 organic compounds and mixtures of compounds that were conventionally divided into the following groups: organic solvents, fuels (mixtures of compounds), organic acids (including amino acids), aromatic carbohydrates, alcohols, ketones, phenols, aldehydes, etc. The determining factors to our choice of compounds were first of all odor, toxicity, and their broad distribution in the environment. Ammonium hydrate (ammonium chloride) was the only inorganic compound in our study. However, this compound corresponded to the given requirements, and was therefore tested (Table 1 and Table 2).

**Figure 1:**
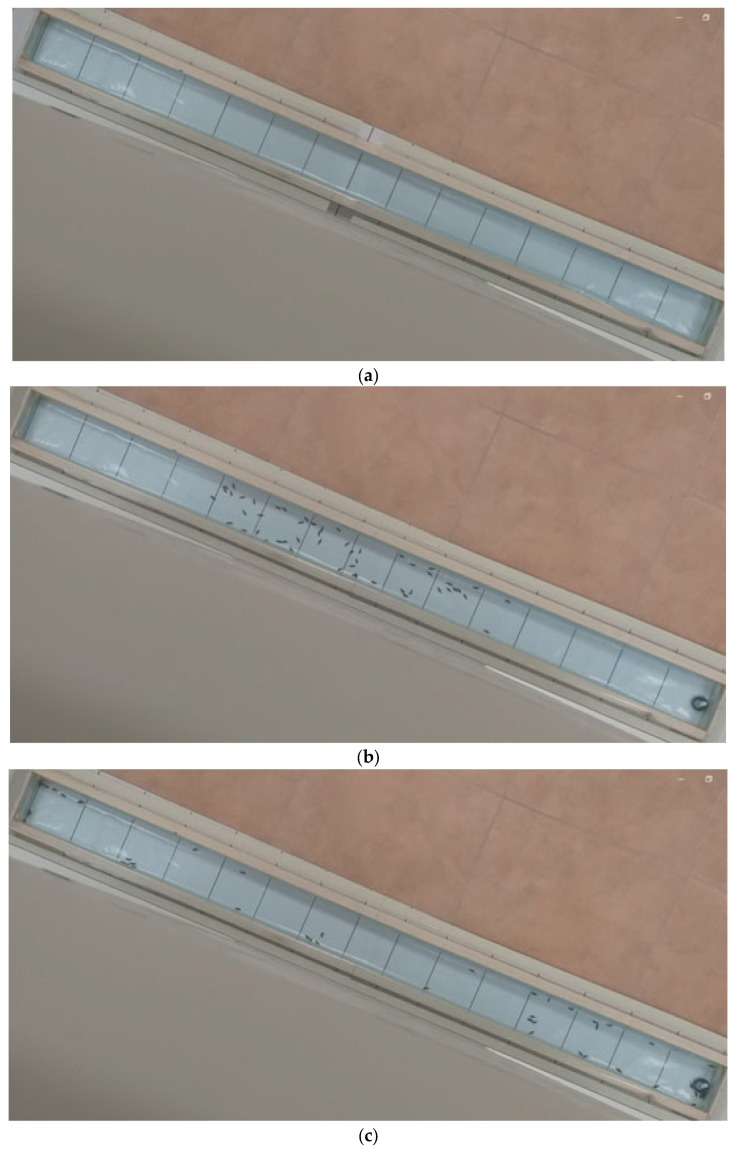
Experimental chamber (**a**) of 150 cm length, 10 cm width, and 10 cm height, divided into 15 10 cm sections (at the bottom of the structure we put polyethylene for each experiment, on the top the chamber is covered by two 80-cm-long glass panels; we released 50 beetles at the beginning of each experiment onto the 8th 10 cm section through opening the glass panels that would then be tightly closed again), location of 50 imagines of on the 30th section (**b**) and 105th section of the experiment (**c**): odor source (cotton swab, moistened with an organic compound) was placed on the first section, in the right lower corner of the photo; a video camera was placed at the height of 150 cm above the experimental chamber; the light of the floor and walls, color of all surfaces on the right and left of the experimental chamber was the same (this is important since beetles have a negative phototaxis).

All the compounds and mixtures used in the experiments were separately tested on the males and females of *Ph. decorus*. While analyzing the footage every 15 s, we paused the video and counted the number of specimens in all the 10 × 10 cm squares of the experimental field (Figure 1). Each video lasted for over 6 min (22 freeze frames). Because imagoes of *Ph. decorus* are very mobile (the beetles can run several dozen centimeters on an even surface in one second), their distribution on the experimental field changed very fast. That is why the 15 s time interval between the pauses was optimal for this species. For less mobile insects in other experiments we used the time interval of 20–60 s. To prevent an adaptation of the olfactory receptors of the beetles to the organic compounds, we performed the experiment with each set of males and females (50 specimens) once a day. 

Unfortunately, in the natural conditions, it is impossible to quickly identify a varying concentration of volatiles at different distances from the odor source due to convectional air flows. Therefore, the results of our laboratory experiments would be hard to replicate in field conditions, since in the experiment we tried to completely eliminate the air-convection factor. At the same time, concentration of volatile molecules in the experimental chamber is perhaps inversely proportionate to the second exponentiation of distance from the odor source. In absence of convection, the dose of the compound is practically irrelevant (for example, 1 or 10 drops). Since the evaporation occurred from a cotton swab, the amount of evaporating molecules was proportionate to the second exponentiation of the cotton swab’s radius, while mass of molecules was proportionate to the third exponentiation of the cotton swab’s radius. Therefore, the effects of mass of a volatile on its concentration in air decreases: the compound’s surface area has a greater effect than mass. 

The obtained data were incorporated into an electronic table for further mathematical analysis. The results were statistically analyzed through a set of Statistica 12.0 (StatSoft Inc., Tulsa, OK, USA). The attractant index was estimated as the ratio of number of beetles on four 10 × 10 cm squares that were the closest to the odor source to the number of beetles on the 4 squares of the experimental field which were the remotest from the odor source.

In this experiment, according to the scale we developed, we took into account ratio of number of insects on 4 squares 10 × 10 cm of the migratory field that were closer to the odor source to the number of insects that were on 4 квaдpaтax 10 × 10 cm of the experimental field that were the farthest from the odor source. In the experiment, according to the scale we developed, we accounted for the ratio of number of insects on 4 10 × 10 cm squares of the migratory field that were closer to the odor source to the number of insects that were on 4 10 × 10 cm squares of the experimental field that were the farthest from the odor source. The attractiveness of a compound was evaluated according to the criteria following scale: attractant index within <0.124—very strong repellent activity, 0.125–0.249—strong repellent activity, 0.250–0.499—repellent activity, 0.500–1.999—absence of activity, 2.000–3.999—attractant activity, 4.000–7.999—strong attractant activity, >8.000—very strong attractant activity (Table 3). 

According to 22 experimental indices of attractiveness for each separate freeze frame, we estimated the mean and its standard error (x ± SE). The data about the effect of a compound or mixtures of compounds on the males and females were compared using ANOVA (Table 4). 

## 3. Results

The tested organic compounds and mixtures caused certain movement reactions in *Ph. decorus*. Some compounds provoked ambulatory reactions, attracting the insects, while others repelled them. The reactions of the males and females to the same compounds or their mixtures most often varied. 

The organic acids in most cases were characterized by the repellent or neutral actions towards the staphylinds (Table 4). The exceptions were amino acids. High repellent activity against both sexes of *Ph. decorus* was exhibited by oleic acid. Acetic acid was a strong repellent against the females and weaker against the males. Oxalic and citric acids exerted moderate (average) repellent activity against the females of *Ph. decorus*, and formic acid against the males. Phosphoric, tartaric, boric, and ascorbic acids caused no effect on the ambulatory activity of the specimens of either sex. The average attractant activity was seen only for some amino acids: glycerin and cysteine for the males, and phenylalanine for the females. The rest of the amino acids had no effect on the locomotor activity of *Ph. decorus*.

Alcohols of various chemical classes comprised a significant share of the organic compounds we tested in the experiment. Alcohols most often exerted moderate repellent or neutral effects on the imagoes of *Ph. decorus*. Exceptions were butanol-1 (strong repellent of females) and methanol (moderate attractant of females). Isopropyl, isoamyl ethers (for both sexes), ethanol, methanol, butanol-1 (males), and glycerin (females) displayed average repellent effects. Benzyl alcohol and ethylene glycol (on both sexes), ethanol (females), and glycerin (males) had no effect on the ambulatory activity of the staphylinids. Ketones, in the example of acetone, were neutral for both sexes of *Ph. decorus* (Table 4 and Table 5).

Aldehydes, in the example of formalin—an aqueous methane solution of formaldehyde—exerted an average repellent action against the males and had no effect on the distribution of the females. The aromatic carbohydrates—xylol and toluol—had a similar effect on the locomotor reaction of the *Ph. decorus* imagoes. Toluol (for both sexes) and xylol (males) caused moderate repellent action. In the experiments with the females, xylol displayed neutral properties and had no effect on distribution of the insects on the experimental field. During the action of hydroquinone (compound of phenol group), we saw no effects on the distribution of *Ph. decorus*. Saturated carbohydrates of the alcanes class, in the example of hexane, caused opposite effects in the individuals of different sexes of *Ph. decorus*. Hexane was a strong repellent against the males and was neutral to the females. The males were more sensitive to the odors of organic compounds. The experiments with ammonium hydrate showed conflicting results: this was a strong repellent against the females and had no effect on the males (Table 4 and Table 5).

Some compounds or mixtures were characterized by opposite effects on the individuals of different sexes of *Ph. decorus*. For example, diesel fuel repelled the males and attracted the females. In general, the tested mixtures of compounds (organic solvents, fuels) did not significantly increase the ambulatory activity of *Ph. decorus*. In most cases, they exhibited a moderate repellent or neutral action towards the insects. Solvent 646 and white spirit insignificantly repelled the individuals of both sexes. Turpentine and gasoline 98 had no effect on *Ph. decorus*. Nefras 80/120, gasoline 95, solvent 649, and diesel fuel had a moderate repellent effect only on the males. We observed no changes in the ambulatory activity of the females of *Ph. decorus* subject to those mixtures (no activity), except for diesel fuel. This type of fuel, in contrast to the other organic mixtures, exerted a moderate attractant activity on the females (Table 4 and Table 5).

## 4. Discussion

According to the literature data, the chemical composition of the protective secretions of the abdominal glands of staphylinids of the subfamilies Omaliinae and Proteininae, analyzed using a gas chromatography–mass spectrometry (GC–MS), includes 98 components, including 46 chemical compounds that were identified as acids, aldehydes, ketone aldehydes, ketones, alcohols, esters, terpenes, aromatic compounds, and carbohydrates. Also, allomones of other staphylinids were found to contain caprylic acid, isovaleric acid, alpha-pinene, beta-pinene, and beta-caryophyllene. In the experiments with larvae of flies *Calliphora vomitoria* (Linnaeus, 1758), aldehydes, ketones, and aromatic compounds used separately exerted repellent effects [21]. 

Over 40 volatile compounds were isolated from the abdominal glands of the Staphylininae subfamily. The secretions of the genera of the Staphylininae subfamily vary in composition; they contain numerous compounds with various structures. The main component of the secretions is iridodial, hence the name of this system of chemical protection of staphylinids—iridodial. It is significantly different from the quinone defense system based on a toxic component [22]. Quinones that are present in allomones, produced by the thoracic gland, are characteristic for staphylinid species of the subfamilies Aleocharinae, Oxytelinae [23]. The main quinones of the defensive secretion of *Aleochara curtula* (Goeze, 1777) are toluquinone and 2-methoxy-3-methyl-1,4-benzoquinone [24]. Allomones of staphylinids of the *Bledius* genus (*B. furcatus* (Olivier, 1811), *B. tricornis* (Herbst, 1784), *B. dissimilis* Erichson, 1840 et al.) contain n-toluquinone and its precursor n-toluhydroquinone, dissolved in a variety of solvents—alkenes, lactones, carbonic acids. Also, besides quinones, diisopropyl and dibutyl ethers were isolated. In total, those compounds repelled small predatory arthropods [25,26]. 

Some components (S-ipsdienol, S-cis-verbenol, 2-methyl-3-buten-2-ol) of aggregational pheromones of bark beetles were attractive for the staphylinids *Philonthus* sp., *Bledius* sp., *Conosoma* sp., *Syntomium aeneum* (Muller, 1821), *Tachinus rufipes* (Linnaeus, 1758), *Tachyporus* sp., *Tyrus mucronatus* (Panzer, 1803), and *Xantholinus* sp. [27]. A similar effect was also seen for R-sulcatol (pheromone of beetles of the *Gnathotrichus* genus). This compound with a rose scent attracted the staphylinids *Nudobius lentus* (Gravenhorst, 1806), *Quedius tenellus* (Gravenhorst, 1806), *Q. xanthopus* (Erichson, 1839), *Anthophagus omalinus* Zetterstedt, 1828, *Hapalaraea melanocephala* (Fabricius, 1787), and *Trimium brevicorne* (Reichenbach, 1816) [28]. Fungal alcohol or 1-octen-3-ol is produced by some plants and fungi. It also forms during the oxidation of linoleic acid. Other than blood-sucking insects of the Diptera order, octenol attracted some species of Staphylinidae (*Atheta* sp., *Euplectus* sp., *Lordithon lunulatus* (Linnaeus, 1760), and some of the *Philonthus* genus (*Ph. succicola* Thomson, 1860). The small staphylinids *Atheta* sp., which live in fungi, were attracted by fatty acids octan-1-ol and nonan-1-ol [29]. Glycerol attracted the mosquito *Aedes albopictus* (Skuse, 1895) [30]. Ethyl alcohol is an attractant of many coleopterans of the families Cerambycidae, Curculionidae, Scarabaeidae, Buprestidae, Silphidae, and Carabidae [31,32]. Methanol exerted attractant properties towards the coleopterans *Callosobruchus chinensis* (Linnaeus, 1758) and *Hypothenemus hampei* (Ferrari, 1867). The female staphylinids of *Ph. decorus* were observed to have the same reaction to this alcohol. Hexane is known as an attractant of some coleopterans, for example, bean weevils (Bruchinae, Chrysomelidae) [33]. In our experiments, we saw an opposite effect. Hexane exerted a strong repellent effect against male *Ph. decorus*. According to the literature data, cantharidin attracted the staphylinids *Diartiger fossulatus* Sharp, 1883, and *Acrotona* sp. It is an oily compound of the terpenoid class, produced by many blister beetles [34]. 

Formaldehyde is an attractant of the ground beetles *Carabus problematicus* Herbst, 1786, *Autocarabus cancellatus* Illiger, 1798, *Pseudoophonus rufipes* (DeGeer, 1774), *Dolichus halensis* (Schaller, 1783), *Pterostichus melanarius* (Illiger, 1798) and staphylinids *Drusilla* sp., *Philonthus* sp., and *Tachyporus* sp. The presence of formaldehyde in the natural conditions is related to the process of burning and breakdown of organic compounds and materials [35]. 

In our experiments, oleic acid caused a strong repellent activity against both sexes of *Ph. decorus*. However, according to some literature data, this compound can be an attractant of ground beetles (*Bembidion obtusidens* Fall, 1922), skin beetles (*Trogoderma granarium* Everts, 1899), ants (*Atta mexicana* (Smith, 1858)), and some species of oribatid mites (Oribatida sp.) [36,37,38,39]. 

Formic acid is one of the main components of protective secretions of the pygidium of many ground beetles of various subfamilies (Anthiinae, Platyninae, Harpalinae, Carabinae, Pterostichinae et al.) [40]. Acid-containing allomones of coleopterans repel natural enemies and competitors for food, as was confirmed by our studies. Formic acid exerted a moderate repelling activity against the male *Ph. decorus*. However, the scientific literature contains data that blood-sucking dipterans (*Aedes aegypti* (Linnaeus, 1762)) were attracted to this compound [41]. 

Similarly to formic acid, acetic acid is a repellent produced by the glands of many ground beetles. This compound was observed to attract a number of dipterans, lepidopterans, hymenopterans, and neuropterans [42,43,44]. 

The main components of the defense mechanism of leaf beetles of the *Chrysochus* genus are amino acids such as phenylalanine, tryptophan, leucine, and diacetyl putrescine [45]. Some amino acids were observed to have attractant properties [46]. For example, L-tryptophan attracted the predatory neuropterans *Chrysoperla carnea* (Stephens, 1836) [47]. In our experiments, amino acids either had no effects on the migratory reactions of the staphylinids or exerted moderate attractive activity (glycine and cysteine for the males, and phenylalanine for the females of *Ph. decorus*).

Biogenic amines and phenolic compounds are present in allomones of caterpillars of the Saturniidae lepidopterans. Those compounds repelled some species of ants [48]. In our experiments, hydroquinone (phenols class) was neutral to the predatory beetle *Philonthus*. It is possible that a crucial factor to the chemical defense of insects is the overall amount (mixture) of secreted compounds. 

In our experiments, aromatic carbohydrates (toluol and xylol) showed weak repellent properties against *Philonthus* sp. However, studies revealed that those compounds can fend off some lepidopteran pests (*Diaphania nitidalis* (Stoll, 1781)) [49]. We had the same results in the experiment with some aromatic-carbohydrates-containing organic mixtures. Gasolines contained up to 16% of aromatic carbohydrates. Moderate repellent effects were exerted by those types of fuel only against the males of *Ph. decorus*. However, the literature has data that xylene attracted the fruit fly *Bactrocera oleae* (Rossi, 1790) which damages fruits of olive trees and also some species of capsid bugs of the *Adelphocoris* genus [50,51]. The percentage of aromatic carbohydrates in diesel fuel is significantly higher (up to 30%). Perhaps this explains the attractiveness of diesel fuel for the females of *Ph. decorus*. 

Uncontrolled environmental pollution with organic compounds of various compositions and their impacts on living organisms require more research [52,53]. There is a need for adequate assessment of how various chemical compounds coming from various technogenic sources into the environment affect valuable agricultural and forest species of invertebrates, including staphylinids [54,55]. Our experimental study of the effects of organic pollutants on the common species of staphylinids in complex with other studies allows a more detailed prediction of possible implications of a broad spread of pollutants in the environment and expansion of the knowledge of the chemoecology of insects. 

## 5. Conclusions

*Philonthus decorus* is one of the most widespread species of rove beetles in different types of plant communities in Europe. Specimens of this species are very often exposed to many chemicals used in everyday life, in industry, and in transport. Our study shows for the first time that exposure to many anthropogenic organic pollutants can reduce beetle abundance. It is likely that exposure to organic pollutants may be one of the important factors reducing the number of predatory beetles (including some species of rove beetles) in anthropogenically transformed landscapes.

## Figures and Tables

**Table 1:** Brief characteristics of the tested compounds.

Name of Compounds	Structural Formula	Melting Temperature; Boiling Temperature	LD_50_ (Rat, Oral), mg/kg	Use in Pharmaceutics and Food Industry	Use in Industry, Construction, and Household
Hexane C_6_H_14_	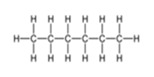	−95.0; 68.0	25,000	Used during extraction of plant oils from peanuts, soybeans, maize, and other oilseed crops	Processing of oil, production of gasoline and additives; chemical industry—fractional purification of compounds, making of chemical reagents, production of glues; tire production; electronics
Toluene C_7_H_8_	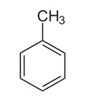	−95.0; 110.6	636	–	In the chemical industry as a raw material for organic synthesis; in the varnish industry (is included in various solvents, varnishes, and paints); as solvent for many organic compounds and polymers; as additive to motor fuels
Xylene (ortho- xylene) C_8_H_10_	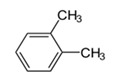	−47.4; 138.5	5000	–	In the oil, chemical, printing, and leather industries, construction (synthesis of terephthalic acid, solvent of varnishes and paints, etc.)
Methanol CH_4_O	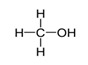	−97.6; 64.7	5628	Used in pharmaceutics	In petrochemical (production of fuel) and chemical industries in making of formaldehyde, acetic acid, plastic, rubber, synthetic fabrics, coloring, fungicides
Ethanol C_2_H_6_O	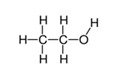	−114.1; 78.2	7060	Used in pharmaceutics as an antiseptic agent, and as a solvent and preservative for medicinal drugs; food additive E1510 (solvent, preservative), is included in alcoholic beverages, dairy products, bakery, and confectionery	Used in chemical industry as a raw material, as a solvent in production of varnishes, household chemicals, component of antifreezes and glass cleaner, etc.; in perfumery and cosmetics
Isopropanol C_3_H_8_O	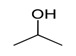	−89.0; 82.6	5045	Used as a disinfectant for external use (substitute of ethyl alcohol); as a raw material for synthesis of acetone, hydrogen peroxide, etc.	Used in industry for metal polishing, maintenance of office equipment; included in cosmetics, perfumery, household chemicals, and maintenance of vehicles
1-Butanol C_4_H_10_O	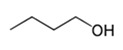	−89.8; 117.7	790	–	In the chemical industry for synthesis of many organic compounds; in the paint and varnish industry; in production of resins and plastifiers; as vehicle fuel
Isoamyl alcohol C_5_H_12_O	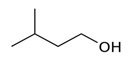	−117.0; 131.1	1300	Used in food production and production of fruit essences	Production of varnishes and paints, plastifiers, as solvent of fats, waxes, resins, and oils, in cosmetics and perfumery
Benzyl alcohol C_7_H_8_O	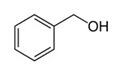	−15.2; 205.3	1230	Used in pharmaceutics as an antimicrobial agent in oil solutions; as food supplement E1519	In the chemical industry as a solvent of varnishes; in perfumery
Hydroquinone C_6_H_6_O_2_	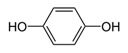	172.0; 287.0	302	Used in production of medicinal drugs; food industry as an antioxidant	In production of organic colorings, photographic materials; in cosmetics
Ethylene glycol C_2_H_6_O_2_	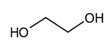	−12.9; 197.3	4700	Used as a cryoprotectant, is included in solutions which preserve the tissues and organs from destruction during thawing	Used as a thermal carrier in household heating and cooling systems of computers; is included in antifreezes and brake fluids; production of many polymers; as solvent of paints; organic synthesis; production of preservatives, and others.
Glycerol C_3_H_8_O_3_	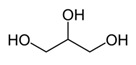	17.8; 290.0	12,600	In pharmaceutics, it is used as a sweetener in syrups and lozenges; in the food industry, it is used as a component of numerous foods (food supplement E422, emulsifier)	Used in the paint and varnish industry; production of plastic; textile, paper, and tanning industries; production of detergents and cosmetics (as humectants, smoothing component); and tobacco production
Formalin CH_2_O	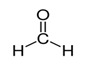	−92.0; −19.0	100	In pharmaceutics, it is used as a disinfectant; in some medicinal drugs	Used in metallurgy; in the oil and petrochemical industries; in the chemical industry—production of formaldehyde resins, antiseptics, solvents for varnishes and paints; as a raw material in organic synthesis; in the paper, textile, and tanning industries; production of agricultural pesticides
Acetone C_3_H_6_O	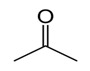	−94.9; 56.1	5800	Used in production of medicinal drugs; for isolating plant extracts from plants; production of bandages; food industry to extract fats, etc.	In the chemical industry it is used as a raw material for synthesis of many organic compounds; production of varnishes and paints; as a surface-cleaning solvent; in the textile industry, perfumery
Formic acid CH_2_O_2_	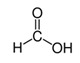	8.4; 100.8	1100	In pharmaceutics it is used as a disinfectant of equipment, to prepare solutions of performic acid; food supplement E236 (preservative)	In the chemical industry it is used as solvent; in textile and tanning industries; in galvanization for metal cleaning; in construction to improve the properties of concrete, etc.
Acetic acid C_2_H_4_O_2_	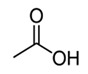	16.0; 118.0	3310	Is a component of medicinal drugs used in dermatology; food supplement E260 (preservation and flavoring)	Production of plastic, varnishes, paints, solvents, etc.
Oleic acid C_18_H_34_O_2_	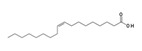	16.3; 360	25,000	Is a component of cosmetics and prophylaxis skin-care products; is present in natural oils and food products	Is used as emulsifier for mechanical treatment of metals, production of varnishes and paints; in soap-making, and other spheres
Oxalic acid C_2_H_2_O_4_	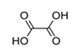	189.0; –	7500	Used in pharmaceutics during production of antimicrobial drugs	In the chemical industry for producing colorings, production of household chemicals; in textile and tanning industries; metal processing; in production of cosmetics as a component of bleaching creams, etc.
Tartaric acid C_4_H_6_O_6_	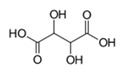	169.0; 399.3	2000	Used in pharmaceutics; in the food industry as a pH regulator and antioxidant in production of canned foods, confectionery, beverages (supplement E334); in winemaking	Used in the textile industry for dyeing of fabric; in cosmetics for production of creams and lotions
Citric acid C_6_H_8_O_7_	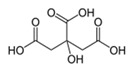	156.0; 310.0	6730	Used in pharmaceutics; as supplement E330—pH regulator and preservative; in production of processed cheese, confectionary, beverages	Used in oil mining during drilling wells; in construction as an additive to cement; in the chemical industry during production of household chemicals, etc.
L-Ascorbic acid C_6_H_8_O_6_	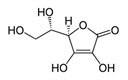	190.0–192.0; –	11,900	A component of medicinal drugs and bioadditives (prophylaxis and treatment of vitamin C deficiency); food supplements E300–E305 (antioxidants, stabilizers, pH regulators)	Used as a powerful reducing agent in the development of film and prints; in cosmetics as a component of creams, etc.
Glycine C_2_H_5_NO_2_	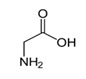	233; –	7930	A component of medicinal drugs and bioadditives as a restorative and sedative agent, for treatment of pathologies of the nervous system; one of the 20 essential amino acids that participate in synthesis of proteins in foods; food supplement E640; ingredient of sports nutrition products	Used in the chemical industry as an additive to galvanizing liquids and a pH regulator
L-Alanine C_3_H_7_NO_2_	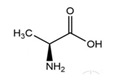	258.0; –	>5110	A component of some medicinal drugs and bioadditives; one of the 20 essential amino acids—monomers of proteins in foods	–
L-Cysteine C_3_H_7_NO_2_S	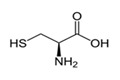	240.0 –	1890	A component of drugs for treatment of pathologies of the respiratory system and the liver; non-essential amino acid present in foods; food supplement E920 (production of bakery and meat products, in sport diet)	Used in cosmetics as the main ingredient of hair-care preparations
L-Asparagine C_4_H_8_N_2_O_3_	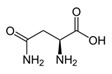	234.0; 438.0	–	A component of some drugs and bioadditives; one of the 20 essential amino acids—monomers of proteins in foods	Used in the chemical industry
L-Arginine C_6_H_14_N_4_O_2_	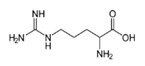	222.0; 368.0	5110	Used in pharmaceutics for drugs for treatment of pathologies of the cardiovascular system; basic amino acid in proteins in foods; essential for children	–
Phenylalanine C_9_H_11_NO_2_	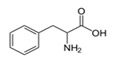	283; _	10,000–16,000	As a component of drugs to treat depressive conditions, neurasthenia, hyperactivity, and others; essential amino acids in foods, food supplement E951 (sugar substitute, in sports nutrition)	–
L-Tyrosine C_9_H_11_NO_3_	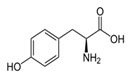	343; –	5110	A component of drugs and dietary supplements, has psychostimulating and antidepressant effects; non-essential amino acid in some foods; ingredient for sport diets	Used in cosmetics, in some preparations for solariums
L-Tryptophan C_11_H_12_N_2_O_2_	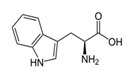	–; –	2000	A component of drugs for treatment of obsessive compulsive disorders, depressive conditions, mitigation of signs of drug withdrawal syndrome; essential amino acid in foods	–
Boric acid BH_3_O_3_	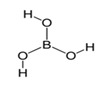	170.9; 300.0	2660	In pharmaceutics as a disinfectant in preparations used in dermatology; supplement E284 (pH regulator and preservative)	In foundries; in production of ceramics, fiber optics, glass; in households (drugs against synanthropic insects)
Ammonia solution NH_4_OH	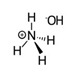	−57.5; 37.7	350	In the food industry (food supplement E527)	Used in agriculture (nitrogen fertilizers)
Phosphoric acid H_3_PO_4_	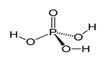	42.3; 212.0	1530	Used as a pH regulator in gassed beverages; for phosphatization of meat; food supplement E338; used in stomatology and orthodontics as an etching solution	Used in freons in industrial refrigerators; as a flux during soldering of metals; as an anti-corrosive agent for treating metals; in aviation industry, etc.

**Table 2:** Short characteristics of the studied mixtures of compounds.

Name of Mixture of Compounds	Name of Compounds, Molecular Formula, Percentage in Mixture	Structural Formula	Melting Temperature. Boiling Temperature, °C	Use in Industry, Construction, and Household
Gasoline 95	Aromatic compounds C_n_H_2n−6_ (4–16%) Alkanes C_n_H_2n+2_ (25–61%) Cycloalkanes C_n_H_2n_ (9–71%) Unsaturated compounds C_n_H_2n_ (13–45%) Carbon monoxide CO Nitrogen oxide NO_x_ Sulfur dioxide SO_2_ Aldehyde	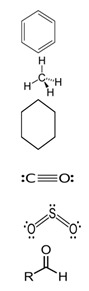	–; 33.0–205.0	Used as fuel in engines of carburetor and injector types, as a solvent in making of paraffin; combustible material
Gasoline 98	Aromatic compounds C_n_H_2n−6_ (4–16%) Alkanes C_n_H_2n+2_ (25–61%) Cycloalkanes C_n_H_2n_ (9–71%) Unsaturated compounds C_n_H_2n_ (13–45%) Carbon monoxide CO Nitrogen oxide NO_x_ Sulfur dioxide SO_2_ Aldehyde	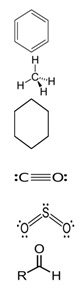	–; 33.0–205.0	Used as high-octane fuel for injector engines with high compression ratio, as a raw material for the petrochemical industry; as a combustible material.
Diesel fuel CnH2n	Alkanes C_n_H_2n+2_ (10–40%) Cycloalkanes C_n_H_2n_ (20–60%) Aromatic compounds C_n_H_2n−6_ (15–30%)	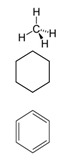	–; 180.0–360.0	Fuel for a large number of freight, passenger, and road transport and special equipment with diesel fuel, can be used for heating systems of private houses, boiler houses; in industry during mechanical and thermal processing of metals, in lubricating and cooling systems
Solvent 646	Ethanol C_2_H_6_O (15%) Butanol C_4_H_10_O (10%) Toluene C_7_H_8_ (50%) Acetone C_3_H_6_O (10% and other)	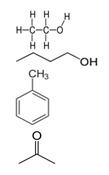	–; 59.0	In petrochemicals and light industry; as a solvent for paints; in production of paints; cleaning of details, removal of dirt; in car servicing; production of perfumery and cosmetics; for household needs
Solvent 649	Xylene C_8_H_10_ (50%) 2-Ethoxyethanol C_4_H_10_O_2_ (30%) Isobutanol C_4_H_10_O (20%)	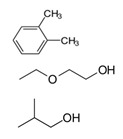	–; –	In the chemical industry; to adjust various paints and varnished to needed viscosity (nitrocellulose paints, varnishes, enamels, coatings)
White spirit	Aliphatic compounds Aromatic compounds ≤16% (can contain ethylbenzene (C_8_H_10_)—0.1–1%) Sulfur (S)—≤0.025%	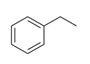	−95.0; 140.0–200.0	Production of paints and varnishes and drying oils; in the road-vehicle industry as a solvent for car services, for degreasing of surfaces, car putties; production of roofing materials
Nefrasas 80/120	Cycloalkanes C_n_H_2n_ N-alkanes C_n_H_2n+2_ Isoalkanes C_n_H_2n+2_ Aromatic compounds C_n_H_2n−6_ (0.5–2.5%)	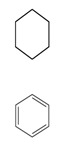	–; –	Used in the chemical, petrochemical, forestry, and textile industries; as a solvent of paints, cleaning of details, removal of coatings, and dirt; for household needs
Turpentine	Turpentine *Pinus sylvestris* L.: Pinene C_10_H_16_ (78%) Delta-3-carene C_10_H_16_ (10–18%) Dipentene C_10_H_16_ (4–6%)	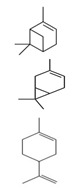	−55.0; 154.0	In the chemical industry (production of camphor, terpin hydrate, and other); varnishes and paints, textile industry; perfumery

**Table 3:** Categories of repellent and attractant effects of compounds on the insects of influence of organic compounds and their mixtures on the locomotor activity of the insects.

Ratio of Number of Insects on 4 10 × 10 cm Squares of the Migratory Field That Were Closer to the Odor Source to the Number of Insects That Were on 4 10 × 10 cm Squares of the Experimental Field That Were the Farthest from the Odor Source	Threshold Value of the Attractiveness Index	Range of the Attractiveness Index	Attractant/Repellent Category
16:1	16.000	>16	extremely high attractant activity
8:1	8.000	8.000–15.999	very strong attractant activity
4:1	4.000	4.000–7.999	strong attractant activity
2:1	2.000	2.000–3.999	moderate attractant activity
1:2	0.500	0.500–1.999	no activity
1:4	0.250	0.250–0.499	moderate repellent activity
1:8	0.125	0.125–0.249	strong repellent activity
1:16	0.062	0.063–0.124	very strong repellent activity
–	–	<0.062	extremely strong repellent activity

**Table 4:** Attraction indices of the tested organic compounds and their mixtures for the males and females of *Ph. decorus* (n = 22; compounds are arranged in the order of increase in significance of differences in their effects on the males and females—see the last column of the table).

Compound	Index of Attraction for Males, Conventional Units, x ± SE	Attractiveness of Compounds for Males	Attractiveness Index for Females, Conventional Units, x ± SE	Attractiveness of Compounds for Females	F, F_0.05_ = 4.07	P
Control	1.224 ± 0.144	no activity	1.248 ± 0.065	no activity	0.02	0.880
Ethylene glycol	0.589 ± 0.037	no activity	0.580 ± 0.036	no activity	0.03	0.855
Solvent 646	0.273 ± 0.042	moderate repellent activity	0.298 ± 0.055	moderate repellent activity	0.13	0.723
Phosphoric acid	1.002 ± 0.196	no activity	0.922 ± 0.099	no activity	0.13	0.717
Toluene	0.368 ± 0.039	moderate repellent activity	0.389 ± 0.036	moderate repellent activity	0.02	0.699
Asparagine	1.889 ± 0.126	no activity	1.763 ± 0.231	no activity	0.23	0.636
Isopropanol	0.485 ± 0.062	moderate repellent activity	0.438 ± 0.074	moderate repellent activity	0.23	0.634
Oleic acid	0.218 ± 0.031	strong repellent activity	0.244 ± 0.023	strong repellent activity	0.47	0.498
Tartaric acid	1.056 ± 0.076	no activity	0.938 ± 0.145	no activity	0.52	0.475
L-Alanine	1.537 ± 0.106	no activity	1.349 ± 0.237	no activity	0.52	0.474
White spirit	0.452 ± 0.074	moderate repellent activity	0.378 ± 0.047	moderate repellent activity	0.72	0.399
Turpentine	0.792 ± 0.147	no activity	0.989 ± 0.101	no activity	1.22	0.275
Nefrasas 80/120	0.468 ± 0.040	moderate repellent activity	0.612 ± 0.097	no activity	1.86	0.180
L-Arginine	1.280 ± 0.100	no activity	1.778 ± 0.324	no activity	2.16	0.149
Acetic acid	0.287 ± 0.057	moderate repellent activity	0.182 ± 0.019	strong repellent activity	2.99	0.091
Acetone	0.632 ± 0.042	no activity	0.512 ± 0.049	no activity	3.50	0.068
Gasoline 95	0.387 ± 0.069	moderate repellent activity	0.602 ± 0.082	no activity	4.03	0.051
Glycine	2.913 ± 0.489	moderate attractant activity	1.828 ± 0.184	no activity	4.31	0.044
Solvent 649	0.385 ± 0.056	moderate repellent activity	0.590 ± 0.080	no activity	4.39	0.042
L-Tyrosine	1.161 ± 0.157	no activity	1.730 ± 0.218	no activity	4.62	0.037
Citric acid	0.537 ± 0.034	no activity	0.424± 0.035	moderate repellent activity	5.43	0.025
Isoamyl alcohol	0.304 ± 0.048	moderate repellent activity	0.465 ± 0.046	moderate repellent activity	5.92	0.019
Gasoline 98	0.511 ± 0.046	no activity	0.745 ± 0.066	no activity	8.49	5.7 × 10^−3^
1-Butanol	0.401 ± 0.071	moderate repellent activity	0.136 ± 0.035	strong repellent activity	11.41	1.6 × 10^−3^
Boric acid	0.505 ± 0.083	no activity	0.921 ± 0.078	no activity	13.34	7.2 × 10^−4^
Phenylalanine	1.695 ± 0.126	no activity	3.868 ± 0.485	moderate attractant activity	18.85	8.7 × 10^−5^
Benzyl alcohol	0.704 ± 0.053	no activity	1.494 ± 0.172	no activity	19.28	7.5 × 10^−5^
Hydroquinone	1.760 ± 0.150	no activity	0.932 ± 0.084	no activity	23.19	1.9 × 10^−5^
Formalin	0.400 ± 0.051	moderate repellent activity	0.720 ± 0.039	no activity	24.38	1.3 × 10^−5^
L-Cysteine	2.443 ± 0.210	moderate attractant activity	1.225 ± 0.125	no activity	24.86	1.1 × 10^−5^
Formic acid	0.382 ± 0.037	moderate repellent activity	0.789 ± 0.072	no activity	25.22	9.9 × 10^−6^
Xylene	0.492 ± 0.098	moderate repellent activity	1.303 ± 0.122	no activity	26.85	5.9 × 10^−6^
Ascorbic acid	0.701 ± 0.043	no activity	1.367 ± 0.111	no activity	31.22	1.6 × 10^−6^
Ammonia solution	0.654 ± 0.061	no activity	0.226 ± 0.041	strong repellent activity	34.47	6.1 × 10^−7^
Glycerol	1.829 ± 0.222	no activity	0.432 ± 0.058	moderate repellent activity	37.60	2.6 × 10^−7^
Hexane	0.242 ± 0.036	strong repellent activity	0.918 ± 0.091	no activity	47.42	2.1 × 10^−8^
Methanol	0.414 ± 0.035	moderate repellent activity	2.604 ± 0.307	moderate attracting activity	50.15	1.1 × 10^−8^
L-Tryptophan	0.429 ± 0.030	moderate repellent activity	1.285 ± 0.106	no activity	60.23	1.2 × 10^−9^
Ethanol	0.371 ± 0.055	moderate repellent activity	1.049 ± 0.058	no activity	71.56	1.3 × 10^−10^
Diesel fuel	0.316 ± 0.070	moderate repellent activity	2.700 ± 0.274	moderate attracting activity	70.88	1.5 × 10^−10^
Oxalic acid	1.168 ± 0.081	no activity	0.274 ± 0.020	moderate repellent activity	113.54	1.6 × 10^−13^

**Table 5:** Effects of organic compounds on the activity of *Ph. decorus*.

Type of Activity	Range of Attractiveness Index	Examples of Compounds or Their Mixtures		Number of Examples, %
		Males	Females	
Very strong repellent activity	0.063–0.124	no	no	0.0
Strong repellent activity	0.125–0.249	oleic acid, hexane	oleic acid, acetic acid, 1-butanol, ammonia solution	7.5
Moderate repellent activity	0.250–0.499	solvent 646, toluene, isopropanol, white spirit, nefrasas 80/120, acetic acid, gasoline 95, solvent 649, isoamyl alcohol, 1-butanol, formalin, formic acid, xylene, methanol, L-tryptophan, ethanol, diesel fuel	solvent 646, toluene, isopropanol, white spirit, citric acid, isoamyl alcohol, glycerol, oxalic acid	31.3
No activity	0.500–1.999	ethylene glycol, phosphoric acid, asparagine, tartaric acid, L-alanine, turpentine, L-arginine, acetone, L-tyrosine, citric acid, gasoline 98, boric acid, phenylalanine, benzyl alcohol, hydroquinone, ascorbic acid, ammonia solution, glycerol, oxalic acid	ethylene glycol, phosphoric acid, asparagine, tartaric acid, L-alanine, turpentine, L-arginine, acetone, nefrasas 80/120, gasoline 95, glycine, solvent 649, L-tyrosine, gasoline 98, boric acid, benzyl alcohol, hydroquinone, formalin, L-cysteine, formic acid, xylene, ascorbic acid, hexane, L-tryptophan, ethanol	55.0
Moderate attractant activity	2.000–3.999	glycine, L-cysteine	phenylalanine, methanol, diesel fuel	6.2
Strong attractant activity	4.000–7.999	no	no	0.0
Very strong attractant activity	8.000–15.999	no	no	0.0
Overall	–	–	–	100.0

## Data Availability

All data are either published with the manuscript or available on request from the lead author.
